# Desmoplastic melanoma: The role of pure and mixed subtype in sentinel lymph node biopsy and survival

**DOI:** 10.1002/cam4.2736

**Published:** 2019-12-05

**Authors:** Annelien E. Laeijendecker, Mary‐Ann El Sharouni, Vigfús Sigurdsson, Paul J. van Diest

**Affiliations:** ^1^ Department of Dermatology University Medical Centre Utrecht Utrecht University Utrecht The Netherlands; ^2^ Department of Pathology University Medical Centre Utrecht Utrecht University Utrecht The Netherlands

**Keywords:** desmoplastic, melanoma, sentinel node, survival

## Abstract

**Background:**

Desmoplastic melanoma (DM) is an uncommon type of melanoma. Two histological subtypes of DM can be distinguished: pure and mixed (PDM and MDM). We hypothesized that discrimination between these subtypes is associated with sentinel lymph node biopsy (SLNB) status and survival.

**Methods:**

Clinicopathological data from PALGA, the Dutch Pathology Register were retrieved from patients diagnosed with DM in The Netherlands between 2000 and 2014. Clinical and pathological variables were extracted from pathology text files, including pure or mixed desmoplastic morphology. A Cox proportional hazard model was performed for overall and recurrence‐free survival (OS and RFS).

**Results:**

A total of 239 patients with DM were included, representing 0.4% of all primary cutaneous melanoma in The Netherlands. A total of 114 PDM and 125 MDM patients were identified. MDM was significantly associated with positive SLNB status (*P* = .035). In multivariable analysis, age (HR 1.10, 95% CI 1.07‐1.14, *P* < .001) and ulceration (HR 1.98, 95% CI 1.05‐3.75, *P* = .036) were significant predictors for OS. For RFS, mixed subtype (HR 2.72 95% CI 1.07‐6.89, *P* = .035), male gender (HR 2.54, 95% CI 1.03‐6.27, *P* = .043), and Breslow thickness (HR 1.13 per mm, 95% CI 1.05‐1.21, *P* = .001) were significant predictors.

**Conclusion:**

MDM is significantly associated with a positive SLNB status. Mixed subtype is significantly correlated with RFS, but not with OS. The distinction between pure and mixed desmoplastic subtype therefore seems to be of clinical importance.

## Graphical abstract

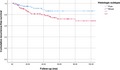

## 1 INTRODUCTION

Desmoplastic melanoma (DM) is an uncommon type of melanoma, representing less than 4% of primary cutaneous melanomas.^1^, ^2^, ^3^, ^4^, ^5^, ^6^ In 1971 Conley et al were the first to describe DM as “an invasive spindle cell tumor with extreme desmoplasia.”^7^ DM has a predilection for male gender and older age. Often, it is found on chronically sun‐exposed areas, most common in the head and neck.^4^, ^8^, ^9^ Breslow thickness is usually greater compared to non‐desmoplastic melanoma (non‐DM), which may be partly explained by the difficulty in diagnosis.^2^, ^8^, ^9^, ^10^, ^11^, ^12^, ^13^, ^14^ DM can easily be confused with other benign or malignant lesions, both clinically and histologically.^3^, ^9^ On histological examination, DM is characterized by the presence of fusiform melanocytes dispersed in a prominent collagenous stroma. DM is often associated with in situ melanoma component, usually lentigo maligna.^3^, ^8^ In 2004, Busam et al distinguished two subtypes: pure desmoplastic melanoma (PDM) and mixed (or combined) desmoplastic melanoma (MDM). DM was defined as pure if the overwhelming majority (≥90%) of the invasive melanoma was associated with prominent stromal fibrosis, and as mixed if the desmoplastic features were combined with densely cellular tumor foci without stromal fibrosis, comprising more than 10% of the entire tumor.^3^ The distinction between PDM and MDM seems to be important because of differences in survival, although this is not yet undisputed.^2^, ^3^, ^12^, ^15^, ^16^ The role of sentinel lymph node biopsy (SLNB) in DM is also controversial. For non‐DM, SLNB is an important and widely used prognostic indicator and staging tool.^1^, ^17^ Due to the lower rates of lymph node metastases compared to non‐DM, some studies suggested that SLNB is not warranted for DM.^1^, ^6^, ^16^, ^18^, ^19^, ^20^ However, others find high enough positivity rates to justify the routine use of SLNB, especially for MDM.^1^, ^2^, ^21^

Therefore, the aim of this study was to determine the yield of SLNB in a Dutch retrospective cohort of patients with DM. Furthermore, we sought to evaluate differences and prognostic indicators for survival between PDM and MDM.

## 2 PATIENTS AND METHODS

### 2.1 Collection of data

Data for this retrospective nationwide study were derived from “PALGA,” the Dutch Nationwide Network and Registry of Histopathology and Cytopathology, that prospectively collects all pathology data from all pathology laboratories in The Netherlands (http://www.palga.nl) since 1991. All data were encoded and used anonymously. Ethical approval was granted by the board of PALGA.

### 2.2 Study population

For this cohort study, data were retrieved from the pathology reports of all newly diagnosed adult DM patients in The Netherlands between 2000 and 2014. Patient with a melanoma without or unclear Breslow thickness were excluded, as well as patients presenting with stage IV disease at the moment of diagnosis. For the present study, this yielded a dataset with histologically proven invasive, primary DMs diagnosed between 2000 and 2014 in The Netherlands.

For each patient, clinical and pathological variables were extracted from the pathology text files, including date of diagnosis, age, gender, Breslow thickness, ulceration (present or absent), subtype of DM (pure or mixed), body site (head and neck, trunk, arms or legs), SLNB enactment (yes or no), SLNB status (positive or negative), and metastases (nodal and distant). Per patient, the first as well as the most advanced category of metastasis was registered. As guidelines do not comment on the time between primary excision and SLNB, in a multidisciplinary setting, we decided to include SLNB performed within 100 days after initial diagnosis.^22^ Regarding pure or mixed etiology, pathology text files were thoroughly examined. If there was no mention of mixed features, it was assumed there was a pure histologic subtype. If this was the case, we noted if expert revision had taken place in order to be sure a mixed subtype was considered as well.

Vital status (dead or alive) for overall survival (OS) was obtained until 1 January 2018 through linkage with the Netherlands Cancer Registry (NCR) hosted by the Comprehensive Cancer Organization of The Netherlands (IKNL). The NCR is a nation‐wide population‐base cancer registry with information on vital status and date of death annually retrieved from the database of deceased persons of the Central Bureau of Genealogy and the municipal demography registries (GBA). Recurrence free survival (RFS) was defined as time to either nodal or distant metastases. For survival analyses, patients with multiple melanomas were excluded.

### 2.3 Statistical analysis

Continuous variables are presented as median with interquartile range (IQR) or mean with standard deviation (SD) for non‐normal distributed data and normal distributed data, respectively. Categorical variables are presented as numbers and percentages and chi‐square test was used to test for significance. Mann‐Whitney test was used to assess significance between non‐normally distributed continuous variables, two‐sample *t* test for normally distributed continuous variables. Kaplan‐Meier curves were generated to assess univariable associations between OS and RFS and desmoplastic subtype. A Cox proportional hazard model was performed for OS and RFS to estimate hazard ratios (HRs) with 95% confidence intervals (95% CI). The proportional hazards assumption was examined by plotting a log‐minus‐log graph for categorical variables. If the lines were parallel, it was assumed that the proportional hazards assumption was not violated. For continuous variables (age and Breslow thickness), Schoenfeld residuals were plotted as a function of time, and a loess curve was fitted. If the curve was horizontal, it was assumed that the proportional hazards assumption was not violated. Variables in the model were: gender, age (continuous), Breslow thickness (continuous), localization, ulceration and histologic subtype. Data were analyzed using SPSS version 26. A two‐sided *P*‐value <.05 was considered significant.

## 3 RESULTS

### 3.1 Patient characteristics

A total of 239 patients with DM were included, representing 0.4% of the primary cutaneous melanoma in The Netherlands between 2000 and 2014. A total of 114 PDM and 125 MDM patients were identified. In the total cohort of DM, 120 (50.2%) patients were male (Table 1). The mean age was 67.2 years (SD 14.2) and the median Breslow thickness was 4.0 mm (IQR 2.6‐7.0). The most common localization was the head and neck (51.9%), followed by the trunk (21.3%). Fourteen patients had multiple melanomas. Follow‐up was not significantly different between PDM and MDM (*P* = .80).

**Table 1:** Baseline characteristics of all patients with desmoplastic melanoma in The Netherlands between 2000 and 2014, stratified for PDM and MDM

	Total (N = 239)	PDM (N = 114)	MDM (N = 125)	*P*‐value
Gender, N (%)				
Female	119 (49.8)	64 (56.1)	55 (44.0)	.061
Male	120 (50.2)	50 (43.9)	70 (56.0)	
Age in years, mean (SD)	67.2 (14.2)	65.6 (15.0)	68.7 (13.3)	.095
Breslow thickness in mm, median (IQR)	4.0 (2.6‐7.0)	4.5 (3.0‐7.0)	3.9 (2.3‐6.0)	.086
Localization, N (%)				
Head and neck	124 (51.9)	60 (52.6)	64 (51.2)	.901
Trunk	51 (21.3)	26 (22.8)	25 (20.0)	
Arms	42 (17.6)	19 (16.7)	23 (18.4)	
Legs	19 (7.9)	8 (7.0)	11 (8.8)	
Missing	3 (1.3)	1 (0.9)	2 (1.6)	
Ulceration, N (%)				
No	150 (62.8)	68 (59.6)	82 (65.6)	.970
Yes	46 (19.2)	21 (18.4)	25 (20.0)	
Missing	43 (18.0)	25 (21.9)	18 (14.4)	
No. of patients, N (%)	62	26	36	
SLN status				.035*
Negative	56 (90.3)	26 (100)	30 (83.3)	
Positive	6 (9.7)	0 (0)	6 (16.7)	
Mixed subtype with	NA	NA		NA
Superficial spreading melanoma			20 (16)	
Lentigo maligna melanoma			47 (37.6)	
Not defined			58 (46.4)	
Follow‐up in months, median (IQR)	41.3 (19.0‐73.6)	36.7 (18.5‐64.7)	43.5 (20.1‐77.3)	.801

Abbreviations: MDM, mixed desmoplastic melanoma; PDM, pure desmoplastic melanoma; SLN, sentinel lymph node.

* Significant.

### 3.2 Sentinel lymph node biopsy

Sixty‐two (25.9%) of 239 DM patients underwent SLNB, with a positive SLNB in 6 (9.7%). When comparing PDM to MDM, no significant differences were found between both groups, except for SLNB status: all six positive SLNBs were of mixed desmoplastic etiology, yielding a significant difference with PDM (*P* = .035; Table 1). Expert revision was performed in 84 (73.7%) patients that were classified as PDM.

### 3.3 Survival analysis

**Figure 1:**
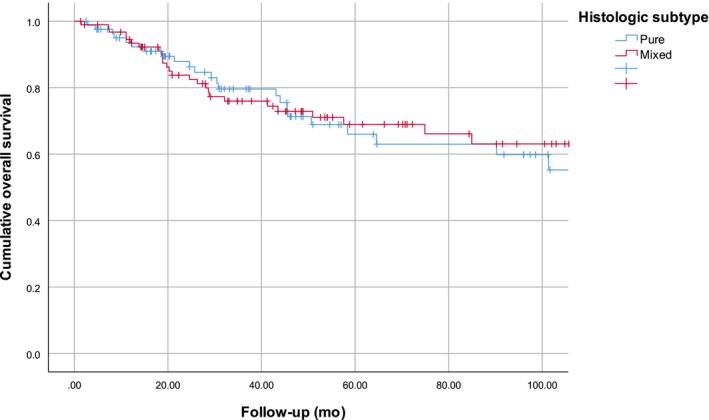
Kaplan‐Meier curves for overall survival stratified for pure desmoplastic melanoma (n = 106) and mixed desmoplastic melanoma (n = 119). (*P*‐value = .80)

**Figure 2:**
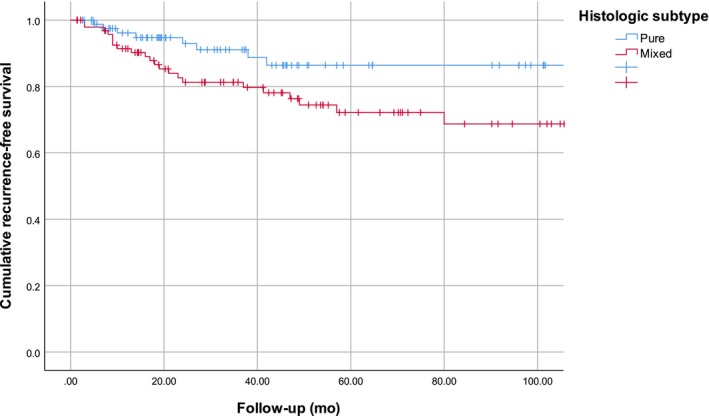
Kaplan‐Meier curves for recurrence‐free survival stratified for pure desmoplastic melanoma (n = 106) and mixed desmoplastic melanoma (n = 119). (*P*‐value = .039)

A total of 8 (7.5%) metastases were found in patients with PDM and 28 (23.5%) in patients with MDM. Five‐year OS was 66.0% for PDM and 69.0% for MDM, yielding no significant difference for desmoplastic subtype (*P* = .80) (Figures 1 and 2). PDM had a 5‐year RFS of 86.4% and MDM of 72.1%. Desmoplastic subtype was significantly associated with RFS in univariable analysis (*P* = .039).

In multivariable analyses, age (HR 1.10, 95% CI 1.07‐1.14, *P* < .001) and ulceration (HR 1.98, 95% CI 1.05‐3.75, *P* = .036) were significant predictors for OS (Table 2). Desmoplastic subtype was not significant (*P* = .970). For RFS, MDM was significantly associated with recurrence (HR 2.72, 95% CI 1.07‐6.89, *P* = .035), together with male gender (HR 2.54, 95% CI 1.03‐6.27, *P* = .043) and Breslow thickness (HR 1.13 per mm, 95% CI 1.05‐1.21, *P* = .001).

**Table 2:** Cox multivariable regression for overall survival and recurrence‐free survival for desmoplastic melanoma in The Netherlands between 2000 and 2014 (n = 149)

	HR	95% CI	*P*‐value
Overall survival^a^			
Age in years	1.10	1.07‐1.14	<.001*
Ulceration			
No	Reference		
Yes	1.98	1.05‐3.75	.036*
Recurrence‐free survival^b^			
Gender			
Female	Reference		
Male	2.54	1.03‐6.27	.043*
Breslow thickness per mm	1.13	1.05‐1.21	.001*
Subtype			
PDM	Reference		
MDM	2.72	1.07‐6.89	.035*

Abbreviations: CI, confidence interval; HR, hazard ratio; MDM, mixed desmoplastic melanoma; PDM, pure desmoplastic melanoma.

a Variables in the model that were not significant: gender, Breslow thickness (continuous), localization, and histologic subtype.

b Variables in the model that were not significant: age (continuous), localization and ulceration.

* Significant.

## 4 DISCUSSION

In this study, we found a prevalence of DM of 0.4% of all primary cutaneous melanoma in The Netherlands between 2000 and 2014. MDM was significantly associated with a positive SLNB status. In multivariable analyses, subtype was associated with RFS but not with OS.

In this study, we found an overall SLNB positivity rate of DM of 9.7% in 62 SLNBs. This is comparable to previous studies with rates ranging from 0% to 18.2%.^2^, ^6^, ^11^, ^15^, ^16^, ^18^, ^20^, ^21^, ^23^, ^24^, ^25^, ^26^, ^27^, ^28^, ^29^, ^30^, ^31^, ^32^ The sample size in these studies varied from 12 to 505 patients who underwent SLNB, which might account for the wide range of SLNB positivity rates found. Five studies found a positivity rate of 0%, but these studies included less than 25 SLNBs.^11^, ^20^, ^27^, ^29^, ^32^ In the systematic review by Dunne et al an overall SLNB positivity rate of 6.5% in 1519 DM patients was found.^1^

When comparing MDM to PDM in the current study, 6/36 (16.7%) of patients with MDM had a positive SLNB and 0/26 (0%) of the patients with PDM (*P* = .0035). In earlier studies with more than 25 SLNBs, positivity rates of MDM ranged from 8.5% to 24.6% and of PDM from 2.2% to 18.2%.^15^, ^24^, ^26^, ^28^, ^33^ All but one study found lower rates for PDM compared to MDM.^24^ When stratified for subtype, Dunne et al found SLNB positivity rates for MDM of 13.8% and for PDM of 5.4%^1^ (no *P*‐value reported). A limited number of studies calculated a p‐value for the difference in SLNB positivity between PDM and MDM. Murali et al found 11/129 (8.5%) positive SLNBs for MDM and 6/123 (4.9%) for PDM in a total of 252 patients who all underwent SLNB (*P* = .25).^2^ Likewise, Conic et al did not find a significant difference between 3/24 (12.5%) positive SLNBs for MDM and 2/11 (18.2%) for PDM (*P* = .31).^24^ Han et al showed in multivariable analysis that after correcting for age, mixed subtype was significantly associated with positive SLNB (OR 3.0) in 205 patients with SLNB, of which 15/61 (24.6%) were MDM and 6/67 (9.0%) PDM.^26^ Lastly, Maurichi et al found a significantly lower positivity rate in PDM (2/49 [4.1%]) compared to MDM (7/51 [13.7%]) (*P* = .022). However, they included not only cases with SLNB but also with elective regional node dissection.^28^

Another interesting and new consequence of a positive SLNB is that it recently has become the gateway to adjuvant immunotherapy for melanoma patients.^34^ The role of immunotherapy in DM was investigated by Eroglu et al., who conducted a retrospective analysis that showed that patients with advanced DM appeared to have higher response rates and favorable clinical outcomes to adjuvant immunotherapy compared to patients with other advanced melanomas, possibly due to a higher mutational load in DM.^35^ One of the proposed criteria to consider adjuvant therapy in melanoma in general is a positive SLNB.^36^ The question remains whether a positive SLNB is a relevant criterion in DM patients for the indication of adjuvant therapy too.

We found a 5‐year OS for MDM of 69.0% and 66.0% of PDM (*P* = .80). Two other studies reported survival percentages for PDM and MDM: Maurichi et al found an OS of 61.3% for MDM vs 79.5% for PDM (*P* < .001) in a total of 242 patients.^28^ Conic et al found an OS of 73% for MDM vs 75% for PDM (*P* = .53) in 58 patients.^24^

Age (HR 1.10, 95%CI 1.07‐1.14, *P* < .001) and ulceration (HR 1.98, 95% CI 1.05‐3.75, *P* = .036) were significantly associated with OS. Posther et al and Han et al also found age as a significant predictor for OS (HR 2.84 and *P* = .021, respectively). Ulceration was included in the analysis in both studies but was not significant.^11^, ^12^ In contrast, Egger et al found ulceration to be significantly associated in an interaction with SLN status (HR 5.96) and age, although in the analysis, not.^21^ Just like the current study, neither Han et al, Wasif et al nor Murali et al found desmoplastic subtype to be a significant predictor in multivariable analysis.^2^, ^12^, ^16^ In a later study by Murali et al MDM was significantly associated with poorer OS (HR 6.17).^33^

Pure desmoplastic melanoma had a 5‐year RFS of 86.4% and MDM of 72.1% (*P* = .039). Pawlik et al also found a better 3‐year disease‐free survival (DFS) for 46 patients with PDM (100%) compared to 19 patients with MDM (78.2%) (*P* = .005),^15^ just like Busam et al (*P* = .01) in 92 patients.^3^ On the other hand, Conic et al found a better RFS for 43 patients with MDM compared to 15 patients with PDM (*P* = .88).^24^ Both Busam et al and Conic et al did not state the exact percentages for PDM and MDM in RFS or DFS. For RFS, we found that MDM (HR 2.72, 95% CI 1.07‐6.89, *P* = .035), male gender (HR 2.54, 95% CI 1.03‐6.27, *P* = .043) and Breslow thickness (HR 1.13 per mm, 95% CI 1.05‐1.21, *P* = .001) were associated with worse RFS. Han et al also found male gender (HR 1.99) to be a significant predictor for worse RFS.^12^ For DFS, Busam et al did report that MDM (*P* = .01) was significant,^3^ just like Pawlik et al (*P* < .001).^15^ Increasing Breslow thickness (HR 1.58) was found to be a significant predictor for worse DFS by Murali et al.^2^

For RFS and DFS in DMs, a wide variety of predictors have been described. This may be due to different definitions of RFS and DFS. We defined RFS as time to either nodal or distant metastasis. Other studies defined RFS or DFS as time to the first recurrence at any site or did not define it all.^2^, ^3^, ^12^, ^15^ Another possible explanation is the difficult histological diagnosis of DM and the variety in definitions for DM used. For example, Busam et al excluded DM with less than 10% desmoplasia in the invasive tumor,^3^ while Murali et al included all melanomas with any degree of desmoplasia.^2^ Lastly, it may be due to the relatively small numbers in these studies. The latter two could also explain the variety in OS.

One of the strengths of this study was that we used a large, nationwide dataset, which positively influences the generalizability of our results. Another strength is the high number of PDM cases in which expert revision took place. Given the rarity of DM, the difficult histological diagnosis and the clinical importance of the distinction between PDM and MDM, evaluation by an experienced dermatopathologist of potential DM cases is most favorable. One of the limitations of our study was the relatively low number of enacted SLNBs. However, most of the other studies that compared SLNB status for PDM to MDM had less total SLNBs performed. Further research should be undertaken to identify other high‐risk factors, besides mixed subtype, for positive SLNB status and to stratify patients that could benefit from undergoing SLNB.

In conclusion, MDM is significantly associated with a positive SLNB status. Mixed subtype is a predictor for RFS but does not influence OS. The distinction between pure and mixed desmoplastic subtype is an important prognostic indicator for DM and continuing making this distinction in future practice is essential.

## CONFLICT OF INTEREST

All authors declare they have no conflict of interest.

## AUTHOR CONTRIBUTIONS

A.E. Laeijendecker contributed to conceptualization, data curation, formal analysis, investigation, methodology, software, visualization, writing—original draft, and writing—review and editing. M.A. El Sharouni contributed to conceptualization, data curation, formal analysis, investigation, methodology, software, supervision, visualization, writing–original draft, and writing—review and editing. V. Sigurdsson contributed to conceptualization, methodology, project administration, resources, supervision, review and editing. P.J. van Diest contributed to conceptualization, methodology, project administration, resources, supervision, review and editing. 

## Data Availability

The data that support the findings of this study are available from PALGA. Restrictions apply to the availability of these data, which were used under license for this study. Data are available from the authors with the permission of PALGA.
